# Lateral Flow Immunoassay Based on Quantum-Dot Nanobeads for Detection of Chloramphenicol in Aquatic Products

**DOI:** 10.3390/molecules28227496

**Published:** 2023-11-09

**Authors:** Qian Han, Ling Fan, Xiuying Liu, Yiwei Tang, Pingping Wang, Zaixi Shu, Wei Zhang, Lijie Zhu

**Affiliations:** 1 School of Food Science and Engineering, Wuhan Polytechnic University, Wuhan 430028, China; hanqian477@163.com (Q.H.); iuhp00@163.com (P.W.);; 2 Key Laboratory for Deep Processing of Major Grain and Oil, Ministry of Education, Wuhan 430028, China; 3 College of Food Science and Technology, Bohai University, Jinzhou 121013, China; lingfan0226@126.com; 4 College of Food Science and Technology, Hebei Agricultural University, Baoding 071001, China; tangyiwei81@163.com

**Keywords:** chloramphenicol, lateral flow immunoassay, quantum dot nanobeads, fluorescent test strip

## Abstract

Quantum dot nanobeads (QBs) were used as signal source to develop competitive lateral flow immunoassay (LFIA) for the detection of chloramphenicol (CAP). The quantitative detection of CAP was achieved by calculating the total color difference (∆E) values of the test line (T line) using the images of test strips. QB-based LFIA (QBs-LFIA) allowed the effective dynamic linear detection of CAP in the range of 0.1–1.5 ng/mL. The limit of detection (LOD) was 3.0 ng/mL, which was 50 and 667 times lower than those achieved for two different brands of colloidal gold kits. The recoveries of CAP during real-sample detection were 82.82–104.91% at spiked levels of 0.1, 0.7, and 1.5 ng/mL. These results indicate that the developed QBs-LFIA facilitates the sensitive detection of CAP.

## 1. Introduction

Chloramphenicol (CAP) is a type of broad-spectrum antibiotic that has been widely used in the treatment of poultry diseases and aquaculture, particularly in the breeding of aquatic animals. It is effective in treating and preventing various bacterial infections and is cost-effective in treating such infections [1,2,3]. However, CAP has serious side effects on human health, such as hypersensitivity reactions, neurotoxic reactions, and leukemia [4,5,6]. Many countries such as USA, France, Germany, and China have implemented bans on the use of CAP in aquatic products [7,8]. However, the illegal use of CAP has continued due to its low cost, and chemical stability [9,10]. Conventional CAP detection methods, including high-performance liquid chromatography (HPLC) [11,12], gas chromatography–mass spectrometry (GC-MS) [13,14,15], and enzyme-linked immunosorbent assay (ELISA) [16], have limitations such as expensive instrumental setup, and time-consuming sample preparation. In addition, if the sensitivity of the method is not sufficient, samples containing trace levels of CAP may be falsely determined as negative results. Therefore, the development of analytical methods for the rapid, effective, and sensitive detection of CAP is of practical significance for maintaining food safety.

In recent years, considering its various advantages such as rapidity, portability, time-efficiency, user-friendliness, and low-cost operation, lateral flow immunoassay (LFIA) has become the most common commercial technique in the areas of food analysis [17], clinical diagnosis [18], and environmental monitoring [19]. The signal source of LFIA plays a key role during the analysis process. Gold nanoparticles have been widely introduced as signal source in conventional LFIA for qualitative or semi-quantitative detection of the analytes. The low sensitivity of the colorimetric readout mode limits the application of conventional LFIA in fields that require highly sensitive and quantitative assays [20]. Instead of gold nanoparticles, various novel nanomaterials, including organic and inorganic dye-doped nanoparticles [21], up-conversion phosphors [22,23], magnetic nanoparticles [24], and fluorescent materials [25,26], have been utilized to improve the sensitivity of LFIA. Among these signal reporters, fluorescence microspheres are most commonly used because of their excellent performance [27,28,29].

Quantum dots (QDs) are one type of the most effective fluorescent source because of their broad excitation spectrum, narrow emission spectrum, high quantum yield, and good stability against photobleaching [30,31,32]. In recent years, the introduction of QDs in LFIA has gradually become a research hotspot. For example, Yuan et al. developed an immunochromatographic strip based on a biotin–streptavidin system to protect antibody activity, using QDs as a signal source to detect aflatoxin B1 in peanut. They achieved a good detection range of 1–10 μg/Kg [33]. In 2023, Hu et al. designed a portable fluorescence immunosensor based on the CdSe/CdS/ZnS QDs for the precise quantification of olaquindox (OLA). The calculated limit of detection for OLA was 0.12 µg/kg in their study [34]. Compared to QDs, quantum dot nanobeads (QBs) are polymer nanobeads consisting of numerous QDs, and exhibit stronger fluorescence intensity and higher tolerance under environmental changes [35,36,37]. The introduction of QBs to LFIA may contribute to further improvement in the stability and sensitivity of LFIA.

In this study, a sensitive competitive LFIA technique based on carboxyl-functionalized CdSe/ZnS QBs (denoted as QBs-LFIA) was designed for the quantitative detection of CAP. The sensitivity, specificity, accuracy, and precision of the developed QBs-LFIA were evaluated by optimizing the detection process, and cross-reactivity. In addition, typical aquatic products were chosen as the test samples to evaluate the developed method.

## 2. Results and Discussion

### 2.1. Principle of QBs-LFIA

**Scheme 1:**
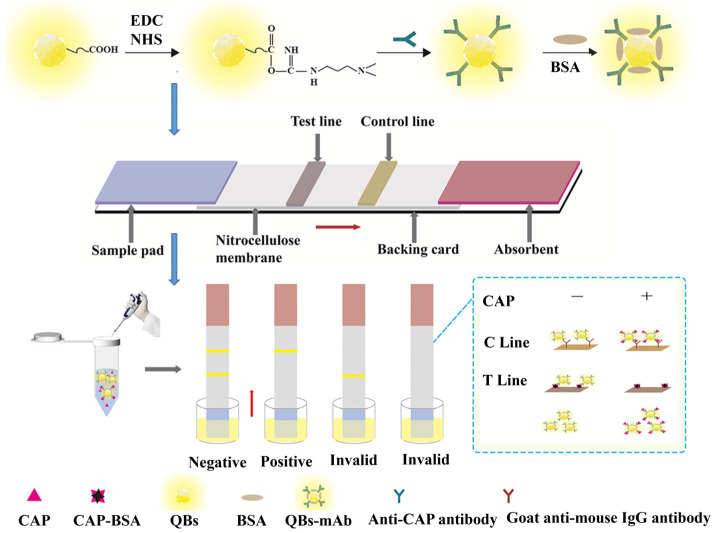
Schematic illustration of QBs-LFIA.

As shown in Scheme 1, QBs were chosen as signal reporters to label mAbs to form QBs-mAb probes. The test was initiated by pre-incubating the sample solution and QBs-mAb probes. After loading the above solution onto the sample pad, all liquids migrated upward. When the CAP content in the sample solution was lower than the threshold, free QBs-mAb was trapped by the CAP-BSA antigen coating at the T line, and the un-trapped QBs-mAb was subsequently captured by the goat anti-mouse IgG at the C line, displaying two yellow fluorescence bands, which indicated negative results. When the content of CAP in the sample solution exceeded the threshold, QBs-mAb was captured, preventing its binding at the T line. This led to a lower intensity of fluorescent band at the T line, indicating positive result for CAP analysis. Thus, the fluorescence intensity of the T line was inversely proportional to CAP concentration. If the C line was not displayed, including the cases where only the T line appears or no bands appear, the strip was considered invalid.

### 2.2. Conjugation of QBs and mAb

**Figure 1:**
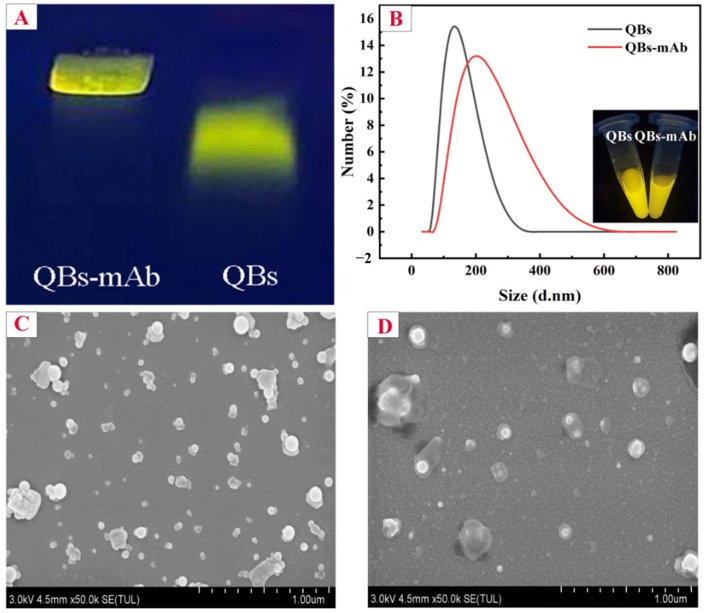
Characterizations of the QBs and QBs-mAb. (**A**) Fluorescence image of QBs-mAb and QBs after gel electrophoresis. (**B**) Hydrodynamic diameter of QBs and QBs-mAb. SEM images of (**C**) QBs and (**D**) QBs-mAb.

The conjugation of the QBs and mAbs was confirmed through gel electrophoresis. In Figure 1A, QBs-mAb conjugates migrated slower than bare QBs, possibly due to the increase in the size of the QBs after conjugation with mAbs. The hydrodynamic sizes, which are referred to geometric dimension sizes, of the QBs and QBs-mAb were analyzed through DLS. After QBs were coated with mAbs, a 57 nm increase in their hydrodynamic size was observed (Figure 1B). The photographs of QBs and QBs-mAb inserted in Figure 1B show that the fluorescence intensity of QBs-mAb slightly decreased in relation to that of bare QBs. These results confirmed the conjugation between the QBs and mAbs.

The SEM results are shown in Figure 1C,D. The QBs appeared to be homogeneously distributed and had relatively uniform spherical microbeads (Figure 1C). After conjugation, an extra layer of material was visible on the surface of the QBs, which was attributed to the coating of mAb over the QBs (Figure 1D).

### 2.3. Optimization of QBs-mAb

**Figure 2:**
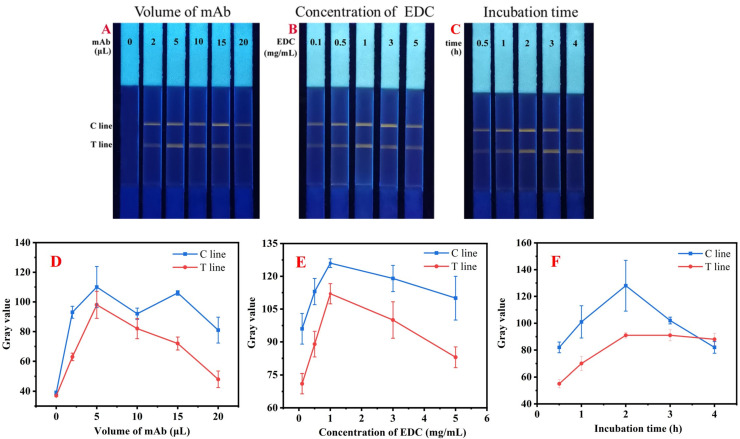
QBs-mAb probe optimization. Fluorescent images of the test strips: (**A**) volume of mAb, (**B**) concentration of EDC, and (**C**) incubation time; gray value analysis: (**D**) volume of mAb, (**E**) concentration of EDC, and (**F**) incubation time.

The conjugation procedure of QBs-mAb was optimized according to the intensity of the T line during the LFIA process. Key factors, including the volume of mAb (1 mg/mL), the concentration of EDC, and incubation time of QBs and mAb, which may affect the sensitivity of LFIA, were optimized. As shown in Figure 2A, the fluorescence intensity of the T line obviously increased with the antibody amount rising from 0 to 5 μL. However, it showed a downward trend with the further increase in the antibody amount. Therefore, 5 μL of mAb was used for the optimal conjugation procedure. The same trend was also observed in the analysis of gray values. In Figure 2D, when the antibody amount was 5 μL, the gray value of the T line reached its maximum at 98 ± 9.07.

The effect of EDC content was monitored to avoid uncontrolled conjugation. The results in Figure 2B show that the fluorescence intensity of the T line reached its peak when the EDC dosage was 1.0 mg/mL, indicating that this was the optimal EDC concentration. Correspondingly, in Figure 2E, the gray values of the T lines initially exhibited an upward trend, and then decreased after the EDC concentration exceeded 1.0 mg/mL.

The incubation time for conjugation was also optimized. In Figure 2C, the fluorescence intensity of the T line stabilized after 2 h. Hence, 2 h was selected. As shown in Figure 2F, the gray values of the T lines increased gradually before 2 h, and became stable after surpassing the incubation time of 2 h. It should be noted that a typical passive adsorption of mAbs onto the surface of the colloidal gold requires 10–30 min of incubation. Comparing to the LFIA based on colloidal golds, the incubation time in this section is a drawback. However, this factor only extends incubation time during probe preparation. It will not impact the detection time and efficiency for the users of the test strips.

### 2.4. Optimization of QBs-LFIA

**Figure 3:**
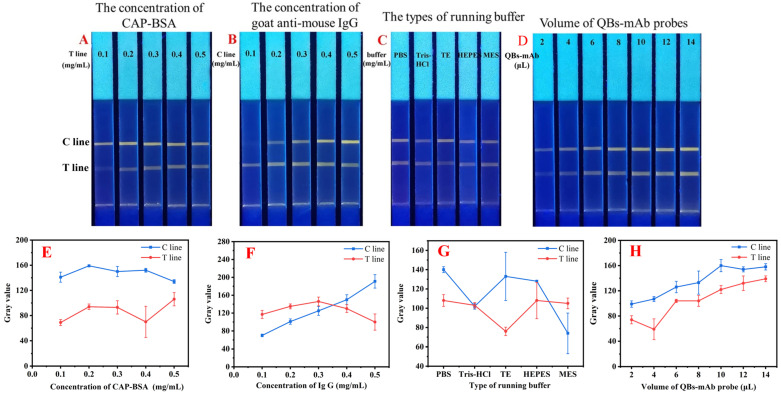
QBs-mAb probe optimization. Fluorescent images of the test strips: (**A**) CAP-BSA concentration, (**B**) concentration of Ig G, (**C**) running buffer, and (**D**) volume of QBs-mAb probes; gray values analysis: (**E**) CAP-BSA concentration, (**F**) concentration of Ig G, (**G**) running buffer, and (**H**) volume of QBs-mAb probes.

To achieve the highest sensing performance of QBs-LFIA, factors including the CAP-BSA on the T line, the coating concentration of goat anti-mouse IgG on the C line, the types of the buffer, and the amount of QBs-mAb probes used for pre-incubation during the detection process were optimized. In Figure 3A, when the concentration of CAP-BSA was 0.3 mg/mL, the fluorescence band of T line was clearly visible. In Figure 3B, the fluorescence of C line was clear and had a similar intensity to T line as the concentration of goat anti-mouse IgG reached 0.3 mg/mL. Considering cost reduction and sensitivity improvement, 0.3 mg/mL of CAP-BSA, and goat anti-mouse IgG was chosen for spraying on the test strips. As shown in Figure 3C, the test strips preparing with PBS as the running buffer exhibited optimal fluorescence intensity for both the T and C lines. Although the fluorescence intensity of the T and C lines using MES and HEPES buffers was similar to that using PBS, the background color was either too dark or too light. Therefore, the PBS buffer was chosen. In Figure 3D, as the volume of QBs-mAb probes increased, the fluorescence intensity of T lines gradually enhanced. Considering cost savings, 6 μL was selected because T line was sufficiently clear.

Correspondingly, the gray values of the test strips were also analyzed, and the results are shown in Figure 3E, F, G and H, respectively. To be noted, the optimal parameters were selected according to both cost savings and the visual appearance of the test strips, rather than solely prioritizing higher gray values. In summary, 0.3 mg/mL of CAP-BSA and goat anti-mouse IgG were selected as the optimal concentrations for coating the T and C lines, respectively. The optimal running buffer was 0.01 M PBS buffer (pH 7.0), and the optimal amount of the QBs-mAb probes was 6 μL.

### 2.5. Analytical Performance of QBs-LFIA

**Figure 4:**
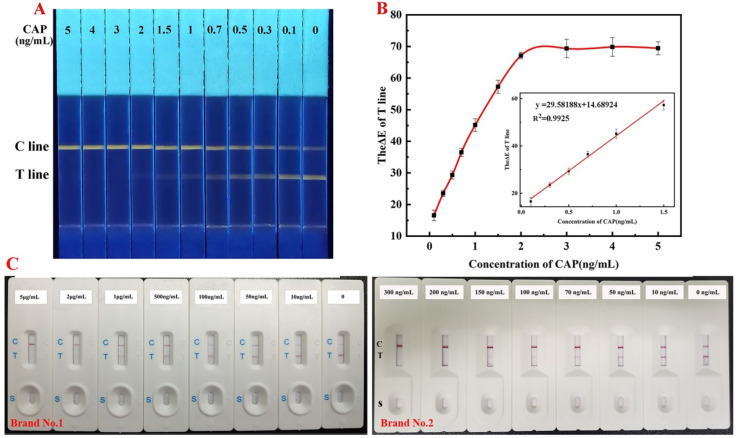
(**A**) Fluorescent image of QBs-LFIA. (**B**) Standard curve for quantitative detection by the LFIA. (**C**) Analysis of sensitivity of different commercial colloidal gold kits.

To determine the sensitivity of QBs-LFIA, spiked samples were prepared and detected under optimal conditions. Fluorescence images of the test strips were captured. As shown in Figure 4A, the fluorescence intensity of the T line gradually decreased with increasing concentrations of CAP and completely diminished at 3.0 ng/mL, indicating a cut-off value of 3.0 ng/mL. Therefore, the visual LOD was obtained as 3.0 ng/mL. In addition, the ΔE values calculated from the T line of the test strip image were plotted against the CAP concentration. In Figure 4B, ΔE of the T line varied linearly with the CAP concentration ranging from 0.1 to 1.5 ng/mL (R2 = 0.9925, *n* = 3), further suggesting that QBs-LFIA can be used to quantitatively detect CAP based on the ΔE values of the test strip image.

The results in this method were compared to those acquired by two different brands of commercial colloidal gold kits. As shown in Figure 4C, the line elimination values of the two commercial test strips were 150 ng/mL and 2 μg/mL, which were 50 and 667 times higher than the results reported in the current study, respectively. This indicates that the current QBs-LFIA is more sensitive than the commercial colloidal gold kits.

We have compared our method with the previously reported methods for CAP detection, and the results are shown in Table S1. Compared to other Immunochromatographic Assays (ICA) based on labels of gold nanoparticles or colloidal gold particles, our QBs-LFIA demonstrated higher sensitivity. It should be noted that the method based on the neutral red probe has the same LOD of 3.0 ng/mL as ours. However, our cut-off value is three times lower than theirs, indicating a more sensitive result for visual detection. It must be acknowledged that some instrumental analysis methods, listed in Table S1, have higher sensitivity than our method. However, these methods require expensive equipment, and higher requirements for technical operators, making them unsuitable for rapid testing.

### 2.6. Determination of Specificity

**Figure 5:**
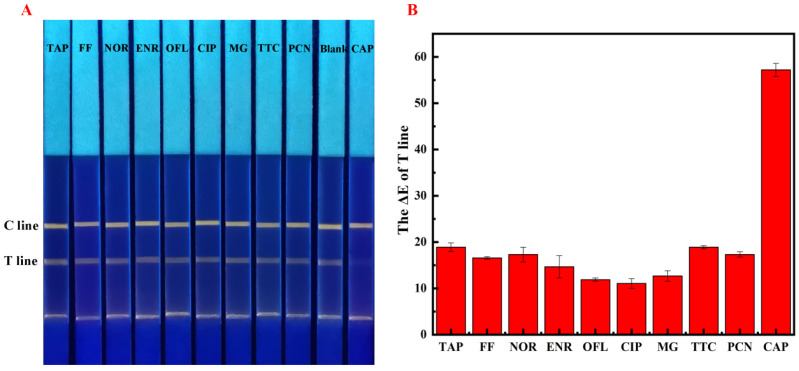
Specificity analysis of the QBs-LFIA. (**A**) Fluorescent image of the QBs-LFIA. (**B**) ΔE values of T line for different analytes.

The specificity of QBs-LFIA was estimated by analyzing structural analogs and other antibiotics that may remain in real samples. As shown in Figure 5A, the test strip used for CAP detection showed no fluorescence at the T line, while bright yellow fluorescence bands appeared at the T lines of the test strips used for detecting the analogs and other antibiotics, indicating the high specificity of QBs-LFIA. In Figure 5B, a high ΔE value was obtained for CAP detection, suggesting that CAP induced noticeable signal inhibition, whereas other structural analogs or antibiotics did not drastically change the visible signal. These results demonstrated that QBs-LFIA was highly specific to CAP.

### 2.7. Analysis of CAP in Real Samples

To test the applicability of the assay developed in this study, different types of aquatic product samples were analyzed. The detection results for the different samples are shown in Table 1. The range of recoveries of CAP detected through QBs-LFIA were 82.82–104.91%, 87.91–96.39%, 88.33–94.93%, and 89.90–93.10% for red drum, grass carp, freshwater shrimp, and scallop, respectively. The coefficient of variation (CV) of all samples at different spiked concentrations range from 5.83% to 9.56%. These results indicate that the current method is applicable for trace CAP analysis in real samples.

### 2.8. Conceptual Products for on-Site Detection

**Figure 6:**
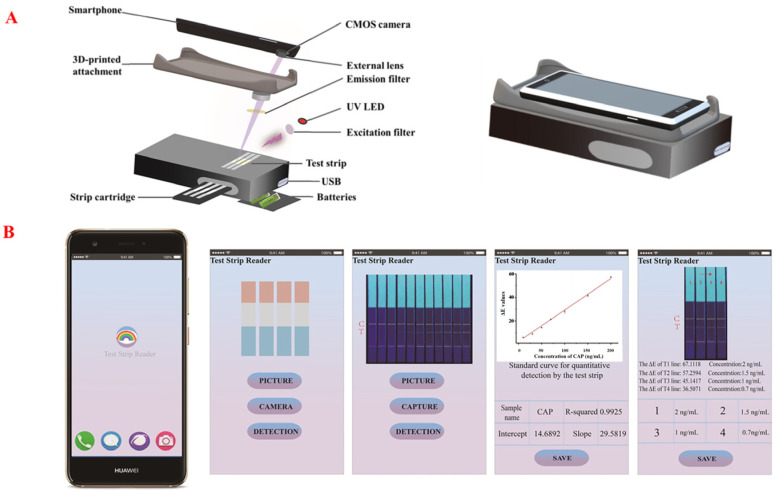
Conceptual design of (**A**) smartphone-based reader and (**B**) data analysis process.

To facilitate the on-site testing of aquatic products, as shown in Figure 6A, we designed a conceptual smartphone-based fluorescence detection device, consisting of a light source, a test strip reader, filters, batteries, and a smartphone. Fluorescence images of the test strips can be captured using a smartphone camera directly. Furthermore, as shown in Figure 6B, the application can calculate the ∆E values of the T lines based on the images, and can automatically plot the calibration curves and determine the concentrations of the target analyte. Although the smartphone readout device is a conceptual product, it serves as a relevant and effective reference for on-site inspection technology.

## 3. Materials and Methods

### 3.1. Materials and Reagents

Carboxyl-functionalized CdSe/ZnS QBs (10 mg/mL, emission occurring at 570 ± 10 nm) were purchased from Shanghai Kundao Biotech (Shanghai, China). The goat anti-mouse immunoglobulin (IgG) antibody, anti-CAP monoclonal antibody (mAb), and corresponding coating antigen (CAP-BSA) were purchased from Shandong Landu Biotech (Jinan, China). CAP, thiamphenicol (TAP), florfenicol (FF), enrofloxacin (ENR), ciprofloxacin (CIP), ofloxacin (OFL), malachite green (MG), norfloxacin (NOR), tetracycline (TTC), penicillin (PCN), 1-ethyl-3-(3-dimethylaminopropyl) carbodiimide hydrochloride (EDC), bovine serum albumin (BSA), N-hydroxysuccinimide (NHS), 2-(N-morpholino) ethanesulfonic acid (MES), and Tween-20 were purchased from Aladdin Chemistry Co., Ltd. (Shanghai, China). Nitrocellulose (NC) membranes, absorbent pads, sample pads, and polyvinylchloride (PVC) backing cards were procured from Shanghai Kinbio Tech Co., Ltd. (Shanghai, China). The commercial colloidal gold kits were purchased from Shenzhen An Kang Testing Technology Co., Ltd. (Shenzhen, China), and Guangdong Dayuan Oasis Food Safety Technology Co., Ltd. (Guangzhou, China). Samples of grass carp (Ctenopharyngodon idellus), red drum (Sciaenops ocellatus), freshwater shrimp (Macrobrachium nipponense), and scallop (Pectinidae) were purchased from a local supermarket in Jinzhou (Jinzhou, China). All samples were packed and stored in a freezer at the time of purchase.

### 3.2. Apparatus

An HM3030 dispenser platform (Shanghai Kinbio Tech Co. Ltd., Shanghai, China) was used to prepare test strips. Scanning electron microscopy (SEM) was performed on a SU8010 microscope (Hitachi, Japan). Dynamic light scattering (DLS) measurements were conducted on a BT-9300ST instrument (Dandong, China). Fluorescence spectra were detected on an F-7000 fluorescence spectrophotometer (Hitachi, Japan). A ZF-7 ultraviolet analyzer was purchased from Shanghai Qinke Analytical Instrument Co., Ltd. (Shanghai, China).

### 3.3. Preparation of QBs-mAb Probes

The surfaces of the QBs were modified with anti-CAP mAbs according to the reported method [38]. Briefly, 2 μL of QBs (10 mg/mL) were dispersed in 400 μL of PBS (0.01 M, pH 7.0); 10 μL of NHS (2 mg/mL) and 20 μL of EDC (1 mg/mL) were added and the mixture was stirred at 25 °C for 30 min to activate the carboxyl groups on the QBs. Subsequently, anti-CAP mAbs (1 mg/mL) were added to the activated QBs at room temperature and the mixture was stirred for 2 h. Then, 100 μL of 1% BSA was added to block the unsaturated sites at 25 °C for 30 min. Finally, the mixture was centrifuged at 4000 rpm for 10 min in an ultrafiltration centrifuge tube (50 kDa) to remove the unconjugated mAbs and BSA. The QBs-mAb probes thus obtained were stored at 4 °C until further use.

### 3.4. Fabrication of QBs-LFIA

The sample pads were pretreated with 0.01 M PBS buffer (pH 7.4) containing 0.5% BSA, 2% sucrose, and 0.05% Tween 20, and then were placed at 37 °C. After drying, the sample pad, NC membrane, and absorbent pad were assembled in a sequence onto the PVC backing cards as shown in Scheme 1. Subsequently, CAP-BSA and goat anti-mouse IgG were applied at a speed of 1 μL/cm, onto the NC membrane to perform T and C lines, respectively. The gap of 5 mm between the two lines was fixed. The test strips were then placed in an oven at 37 °C for 2 h. Finally, the test strips were cut and stored in sealed bags.

### 3.5. Optimization of QBs-LFIA

Firstly, the coating concentration of CAP-BSA on the T line was optimized. The CAP-BSA coating antigen was diluted to various concentrations of 0.1, 0.2, 0.3, 0.4, and 0.5 mg/mL, and sprayed onto the NC membrane as the T line at a rate of 1 μL/cm. Additionally, the C line was coated with goat anti-mouse IgG at a concentration of 0.5 mg/mL. After mixing 5 μL of QBs-mAb fluorescent probe with PBS buffer, the mixture was applied to the test strip. After 10 min of chromatography, the test strip was observed under a 365 nm UV lamp.

To optimize the coating concentration of goat anti-mouse IgG on the C line, the goat anti-mouse IgG was diluted to various concentrations of 0.1, 0.2, 0.3, 0.4, and 0.5 mg/mL. The optimization method for the C line is the same as that for the T line, except that the concentration of the T line is determined as the optimal concentration obtained from the previous optimization step.

CAP-BSA and goat anti-mouse IgG were applied at 0.3 mg/mL, respectively, onto the NC membrane to perform T and C lines. Various buffer including PBS, Tris-HCl, TE, HEPES, and MES were tested. In addition, different volumes of QBs-mAb probes including 2, 4, 6, 8, 10, 12, 14 μL were performed to optimize the amount of QBs-mAb.

### 3.6. Specificity

Structural analogues of TAP, and FF, along with seven interferences including NOR, ENR, OFL, CIP, MG, TTC, and PCN were selected for the specificity test. The concentrations of the structural analogues, and interferences were set at 1 μg/mL, while the CAP concentration was maintained at 3 ng/mL. During the test, the prepared strip was inserted into a mixture comprising 6 μL of QBs-mAb probes and 100 μL of either structural analogues or interference solution at 25 °C. After 20 min, the strip was placed under UV light (365 nm) to observe the detection results.

### 3.7. Sample Preparation and QBs-LFIA Detection

Samples were pretreated according to the previous literature [39]. Briefly, muscle tissues from the samples were homogenized. Ethyl acetate (5 mL) was added to the homogenized sample (1.0 g). After vortexing for 10 min, the mixture was centrifuged (4500 rpm, 10 min). Remove the supernatant to a beaker and repeat the above extraction step thrice. Combine the supernatant and dry them under a nitrogen atmosphere. Then, n-hexane (2 mL) and 0.1 M PBS (pH 7) were added to the above plastic centrifuge tube to dissolve the dried residue. Centrifuged again, and the subnatant was collect as the prepared sample solution. Finally, standard CAP solutions at the concentrations of 10, 70, and 150 ng/mL was diluted 100-fold with the sample solutions, which was considered as spiked samples (at the levels of 0.1, 0.7, and 1.5 ng/mL) for further analysis.

The prepared strip was inserted into a mixture of QBs-mAb probes (6 μL) and sample solution (100 μL) at 25 °C. After 20 min, the strip was placed under UV light (365 nm) to observe the detection results.

For comparison test, the commercial colloidal gold kits were performed according to the user manual recommends.

### 3.8. Data Analysis

CIELAB coordinates (International Commission on Illumination L, a, and b values) were analyzed using Photoshop based on the photographs of the test strips (T and C lines). The total color difference (ΔE) values were processed using the following equation: ∆E = $\sqrt{{\Delta L}^{2}+{\Delta a}^{2}+{\Delta b}^{2}}$, where L is the lightness, a is the deviation from green to red, and b is the deviation from blue to yellow [40].

The gray values were extracted from the black and white versions of the fluorescent images of the test strips, which were processed using Photoshop software 13.0.

## 4. Conclusions

QBs, as an effective fluorescent signal reporter and a promising alternative to conventional nanoparticles, were successfully integrated with LFIA test strips and proposed for the detection of CAP residues. The LFIA test strips could produce noticeable yellow fluorescence during the detection process. Without using a fluorescent test strip reader, the CAP was detected quantitatively by calculating the ∆E values of the T lines based on the images of the test strips. The detection limit was 3.0 ng/mL, and the linear detection range was 0.1–1.5 ng/mL. Under optimal conditions, the sensitivity of QBs-LFIA was improved by 50 and 667 times comparing to two types of commercial colloidal gold kits. The method exhibited good selectivity towards the target analyte in the presence of structural analogs and interferences. Moreover, the applicability of the proposed QBs-LFIA was verified by using it to analyze different aquatic products, including red drum, grass carp, freshwater shrimp, and scallop. The results of these tests validated the promising prospects of the proposed method toward real-sample detection. A conceptual smartphone-based fluorescence detection device was designed to automatically read the results of LFIA strips, with a quantitative analysis application software, demonstrating a promising potential for on-site detection.

## Figures and Tables

**Table 1:** Application of QBs-LFIA to real samples for CAP detection.

Sample	Spiked (ng/mL)	Measured (Mean ± SD, ng/mL)	Recovery (%)	CV (%)
Red drum	0.1	0.10 ± 0.01	104.91	9.56
	0.7	0.65 ± 0.04	92.44	6.86
	1.5	1.24 ± 0.07	82.82	5.83
Grass carp	0.1	0.1 ± 0.01	95.06	6.69
	0.7	0.67 ± 0.04	96.39	6.01
	1.5	1.32 ± 0.10	87.91	7.36
Freshwater shrimp	0.1	0.09 ± 0.01	94.40	8.34
	0.7	0.66 ± 0.04	94.93	5.96
	1.5	1.32 ± 0.12	88.33	9.29
Scallop	0.1	0.09 ± 0.01	89.90	6.21
	0.7	0.64 ± 0.05	91.90	7.43
	1.5	1.40 ± 0.10	93.10	6.91

## Data Availability

Data are contained within the article and Supplementary Materials.

## Supplementary Materials

The following supporting information can be downloaded at: https://www.mdpi.com/article/10.3390/molecules28227496/s1. Table S1: Comparison table of the sensitivity for the CAP detection reported in the literatures. Refs. [41,42,43,44,45,46] are cited in Supplementary Materials.
