# Supplementary material for: Lateral Flow Immunoassay Based on Quantum-Dot Nanobeads for Detection of Chloramphenicol in Aquatic Products
Source: Molecules. 2023 Nov 9;28(22):7496. doi: 10.3390/molecules28227496 (PMC10673565; doi:10.3390/molecules28227496)
Supplement: Supplementary file 1 [file molecules-28-07496-s001.zip › molecules-2651448-supplementary.pdf]

Table S1. Comparison table of the sensitivity for the CAP detection reported in the literatures.

Sample	Method	Label	Sensitivity	References
Milk	Immunochromatographic Assay	Colloidal Gold Particles	LOD 10 ng/mL	[1]
Milk	Immunochromatographic Assay	Gold Nanoparticles	LOD 5 ng/mL	[2]
Milk, and honey	Immunochromatographic Assay	Neutral Red Probe	vLOD 3 ng/mL<br>Cut-off value 9 ng/mL	[3]
Animal tissues and urine	LC-MS/MS	NA	0.02 μg/kg	[4]
	GC-MS	NA	2 μg/kg	
Shrimp and crab meat	HPLC	NA	10 μg/kg	[5]
	GC–ECD	NA	1 μg/kg	
	GCMS–EI–SIM	NA	1 μg/kg	
	GCMS–NCI–SIM	NA	0.1 μg/kg	
Water, milk, and honey	Electrochemical Sensor	Silver Nanoparticles	265.6 nM	[6]
Fish	Immunochromatographic Assay	QBs	LOD 3 ng/mL<br>Cut-off value 3 ng/mL	In this study

References

1. Byzova, N.A.; Zvereva, E.A.; Zherdev, A.V.; Eremin, S.A.; Dzantiev, B.B. Rapid Pretreatment-Free Immunochromatographic Assay of Chloramphenicol in Milk. Talanta **2010**, 81, 843–848.

2. Hendrickson, O.; Zvereva, E.; Shanin, I.; Zherdev, A.; Dzantiev, B. Development of a Multicomponent Immunochromatographic Test System for the Detection of Fluoroquinolone and Amphenicol Antibiotics in Dairy Products. J Sci Food Agric **2019**, 99, 3834–3842.

3. Wang, S.; Du, T.; Liu, S.; Li, Y.; Wang, Y.; Zhang, L.; Zhang, D.; Sun, J.; Zhu, M.; Wang, J. Dyestuff Chemistry Auxiliary Instant Immune-Network Label Strategy for Immunochromatographic Detection of Chloramphenicol. Food Chemistry **2023**, 401, 134140.

4. Gantverg, A.; Shishani, I.; Hoffman, M. Determination of Chloramphenicol in Animal Tissues and Urine. Analytica Chimica Acta **2003**, 483, 125–135.

5. Shen, H.-Y.; Jiang, H.-L. Screening, Determination and Confirmation of Chloramphenicol in Seafood, Meat and Honey Using ELISA, HPLC–UVD, GC–ECD, GC–MS–EI–SIM and GCMS–NCI–SIM Methods. Analytica Chimica Acta **2005**, 535, 33–41.

6. Batish, S.; Rajput, J.K. Quercetin Capped Silver Nanoparticles as an Electrochemical Sensor for Ultrasensitive Detection of Chloramphenicol in Food and Water Samples. Journal of Food Composition and Analysis **2023**, 122, 105421.
